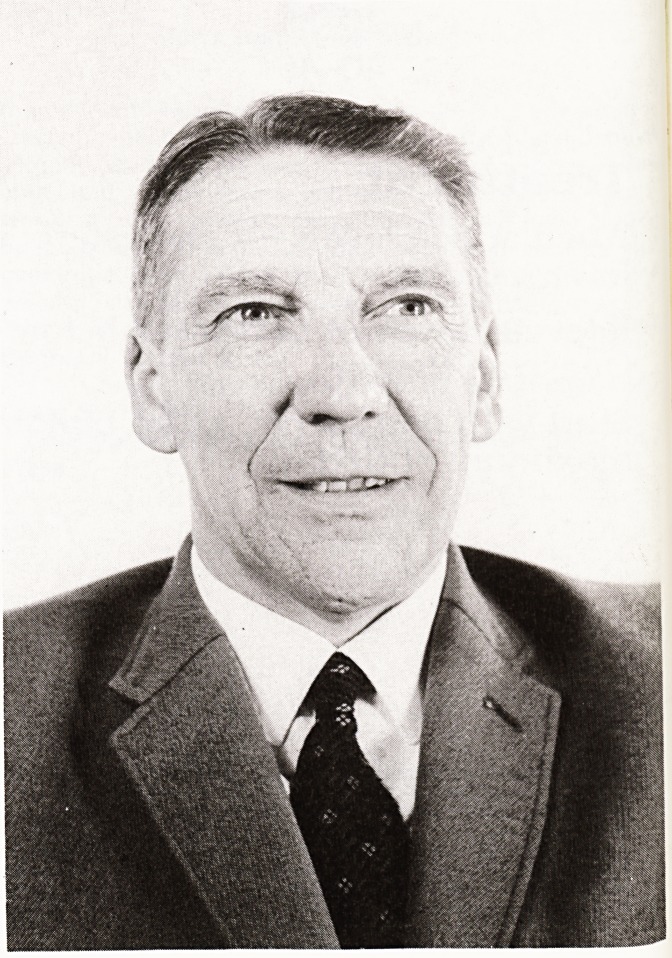# Professor John Clutton Brock

**Published:** 1987-02

**Authors:** 


### Obituary

### Professor John Clutton-Brock, MA, MB, BChir, FFARCS, DA

Professor John Clutton-Brock, the first professor of anaesthesia in the University of Bristol died peacefully on October 13th, 1986.

J.C-B was born in 1912 in Sarncombe, Surrey, the son of an eminent barrister, Arthur Clutton-Brock and his wife Evelyn, a member of the famous Vernon Harcourt family. His education was impeccable—Charterhouse, Trinity College Cambridge and Bart's. He came to the West Country for his first post as a doctor at Bridgwater and then, after a period in the RAMC, went into general practice in Lincoln. In those days the majority of anaesthetics were given by students, housemen or GPs and, for John Clutton-Brock, the subject became a consuming interest and occupied more and more of his time in general practice. Although self taught in the main, clearly he was very good at it and his skills were held in very high esteem for he was soon appointed as one of the first consultant anaesthetists at Lincoln County Hospital, shortly after the introduction of the NHS. A few years later he moved to another Consultant post at Selly Oak in Birmingham. However, the academic urge was always there and when the post of Lecturer in the Department of Anaesthesia at this University became available in 1953 he was appointed to work with the late Ronald Wolmer. Together they created the very sound basis for the present-day flourishing academic department. When Ronnie Woolmer left in 1958 to take up the BOC Chair in Anaesthesia at the Royal College of Surgeons JC-B succeeded him as Head of the Academic Department and when the first Chair in Anaesthesia in Bristol was created in 1965 he was the natural choice for the post which he held with great distinction for 10 years, retiring with the honour of being appointed Professor Emeritus by the University.

He was elected President of The Society of Anaesthetists of the South Western Region for the year 1965-66 and this most successful term of office was notable for the wit of his after dinner speeches and his practical demonstrations of electrical anaesthesia on animals.

JC-B's scientific contributions were always of the very highest standards—here was a man with that inquisitive and innovative nature that is the hallmark of the true researcher. Working with the late Dr. Grey Walter, at both the Burdon Institute and the BRI, he became an expert in electroencephalography and its clinical application. He sought to find that delicate balance between the safety of light anaesthesia and the possibility of awareness during surgery, he revised our thoughts on the barbiturates, he used nitrous oxide with consumate skill, and he anaesthetised goldfish and calves with electricity. With Barbara Weaver and Geoffrey Burton he pioneered anaesthetic techniques suitable for open heart surgery under deep hypothermia in its very early days when it was being undertaken by Bob Horton, Ronnie Belsey and Gerry Keen.

Always his prime interest was in safety in anaesthesia and having observed the errors in our gas flowmeters due to static electrical forces, he was the first to devise a method of overcoming the problem. This is now standard manufacturing practice world-wide today.

JC-B was a prolific inventor possessed of both engineering and electronic skills. He built his own ventilator with features which were well ahead of its time. Alas, marketing talents were not the forte of this modest individual and he never translated his brilliant ideas into mass production. He delighted solely in problem-solving and design and it was left to others to pick up his ideas and inventions and turn them into products for mass usage and application.

JC-B was a benign man, almost invariably gentle in nature, but he would never compromise when he believed he was right and when he thought patients lives were potentially at stake. It took an astute observer with formidable powers of logical reasoning to put the finger immediately on the cause for a patient's unexplained death as probably being due to a contaminated $N_2O$ cylinder. Such a problem had never happened before and the production quality control was thought to be foolproof; however, this quiet but firm man, within hours of the event, had persuaded the combined mights of the British Oxygen Company and the DHSS to withdraw every $N_2O$ cylinder in the land and set about testing them for contaminants. Contaminants they found in a small batch of cylinders and undoubtedly many lives were saved by his prompt and persistent action. Many of us would have prevaricated in the circumstances but JC-B had the courage of his convictions and the strength of character to provoke immediate action from the authorities. The rest of us learned within minutes to give quite adequate anaesthetics without our mainstay agent of over 100 years.

JC-B knew a great deal about virtually everything—the arts, music, history (particularly Egyptology), gardening, motor cars and pottery. As John Bowes[1] wrote in his

"Farewell to the Professor", on the occasion of JC-B's retirement "one of my greatest pleasures was to sit down at lunch with him just to absorb his conversation on any topic which would be discussed so amusingly as to make one forget, briefly, the most depressing realities of every day life".

JC-B was, in truth, an impishly humerous man whose command of the English language was quite formidable. For one of us (PJFB) the first meeting with him was in 1962, across a table during an interview for the post of Registrar at the, then, United Bristol Hospitals. After delving into the applicants rather modest career as an anaesthetist he decided on a personal approach and asked "Have you any children?". The applicant replied that he had not, but feeling this a rather weak response, and wishing to score at least some points from the interview, he added brightly "but I have got a dog". JC-B smiled benignly and said "Well I am sure you will get better results as time goes on".

In addition to many erudite scientific articles, JC-B contributed others that were renowned for their humour for they demonstrated that power of lateral thinking, which is the hallmark of original brilliance. Who else could ponder on Vin de Table and reason logically that other pieces of furniture should be allocated a vintage appropriate to their ambience. In a memorable article in Anaesthesia Points West he suggested Vin de Garde Robe should be selected to be imbibed by a husband patiently waiting for his wife to select the appropriate dress for the occasion and a Vin de Toilette for the wife waiting to complete her make up while her nail polish was drying not to mention a Vin de Loo to occupy those wasted hours of potentially lost drinking time.

JC-B was married to his wonderful wife Joy for 35 years. They were completely devoted to each other and there is no doubt whatsoever that the peace and tranquility that underlined his character and enabled his creative talents to emerge and flourish was in very large measure due to Joy's very selfless love for him. Her own strong and constructive personality enabled them to act in concert and give strength and support at all times to their nearest and dearest.

John leaves five children—a new generation who live to carry on a famous and talented line; Tom is already following impressively in his father's footsteps. They will always be very, very proud of their father as we who were priveledged to know him admired his talents and are grateful for his teaching and delighted in his company.

P.J.F.B. and G.W.B.

**REFERENCES**

1. BOWLES, J. B. John Clutton Brock-Farewell. *Anaesthesia Points West.* 1975; 8: 40 (Autumn)
2. CLUTTON-BROCK, J. Le Vin de Table. *Anaesthesia Points West.* 1986; 19: 48.

## Figures and Tables

**Figure f1:**